# Cardiac Resynchronization Therapy Device Implantation in a Patient with Congenitally Corrected Transposition of Great Vessels

**Published:** 2017-01

**Authors:** Reza Mollazadeh, Masoud Eslami

**Affiliations:** *Imam Khomeini Hospital, Tehran University of Medical Sciences, Tehran, Iran.*

**Keywords:** *Cardiac resynchronization therapy*, *Heart defects/ congenital*, *Congenitally corrected transposition of the great arteries*

## Abstract

A 29-year-old woman was referred to our hospital due to exacerbation in dyspnea on exertion and easy fatigability. A known case of congenitally corrected transposition of the great vessels and congenital complete heart block, she had already received a permanent single-chamber pacemaker. Decision was made to implant a biventricular pacemaker for the treatment of the failing heart. Excellent coronary sinus lead implantation was done, conferring amelioration of symptoms, QRS narrowing in the electrocardiogram, and improvement of systemic ventricular systolic function in echocardiography. Over a 15-month follow-up period, she had no dyspnea on exertion. This case highlights the significance of upgrading pacemakers in patients with heart failure.

## Introduction

Advances in the medical and surgical care of patients with congenital heart disease (CHD) have resulted in a larger proportion of such patients reaching adulthood. Patients with a systemic right ventricle or single ventricle gradually develop symptomatic systolic dysfunction. Cardiac resynchronization therapy (CRT) is one of the best approaches to treating systemic ventricular systolic dysfunction and ipsilateral bundle branch block (native or pacemaker induced) in pediatric and adult patients.^1^^, ^^2^

Given the technical challenges in the implantation of CRT devices, we decided to present this case and discuss the pitfalls.

## Case Report

**Figure 1:**
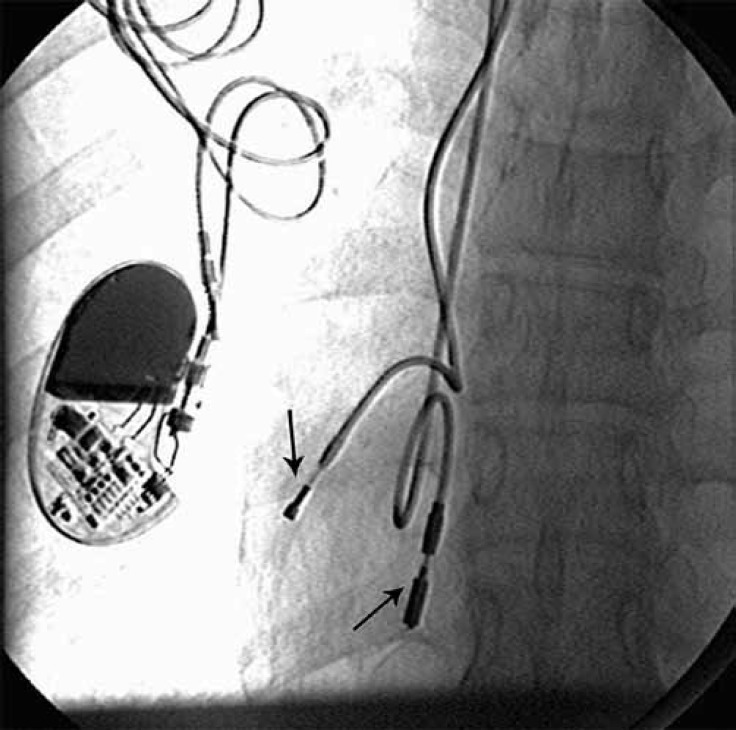
Fluoroscopic anteroposterior view of the chest before the implantation of a cardiac resynchronization therapy device.  There is an old single-chamber permanent pacemaker on the right side. The arrow pointing downward shows a lead implanted 14 years previously, and the arrow pointing upward shows a lead implanted 10 years previously. Both leads are in the right ventricle.

We describe a 29-year-old woman, who was referred to our center for the treatment of gradual exacerbation in dyspnea on exertion and easy fatigability of 6 months' duration. A known case of congenitally corrected transposition of the great vessels (CC-TGV), the patient had undergone single-chamber permanent pacemaker implantation via the right subclavian vein owing to complete heart block 14 years previously. Four years later, there was a rise in the capturing threshold of the right ventricular lead, prompting the treating physicians to implant a new lead (Figure1).

On her referral to our center, she underwent echocardiography, which showed moderate to severe systolic dysfunction of the morphological (systemic) right ventricle. Considering the persistence of the symptoms despite medical therapy, decision was made to implant a CRT device in accordance with the new American College of Cardiology/ American Heart Association 2012 guideline, stipulating that patients with severe systemic ventricular systolic dysfunction and ventricular pacing > 40% of the times be upgraded to CRT.^3^

The well-documented challenges of the implantation of a CRT device from the right side (especially in patients with complex congenital heart disease) led to the decision to abandon the old leads and implant a completely new system from the left side. 

**Figure 2:**
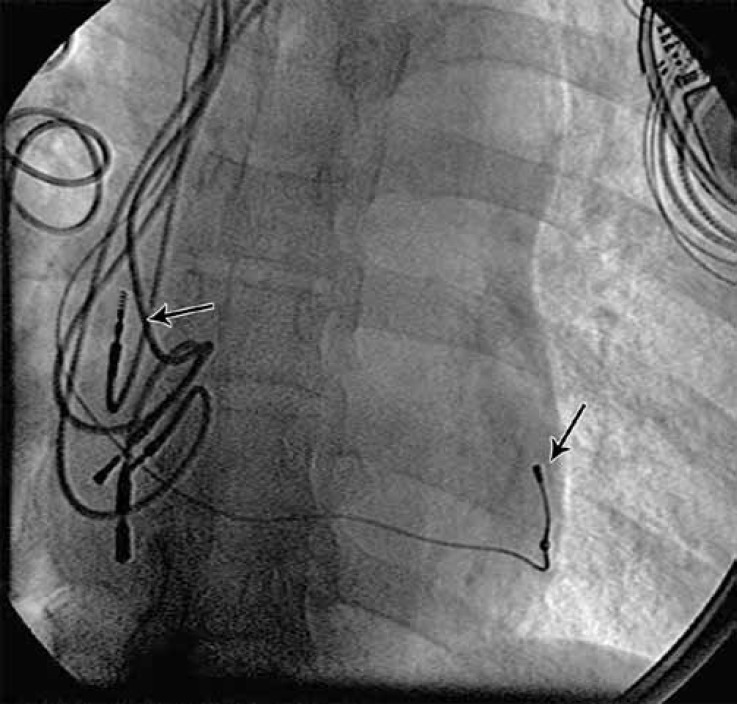
Successful cardiac resynchronization therapy device implantation. The fluoroscopic left anterior oblique view (30°) of the chest. The arrow pointing downward shows the coronary sinus lead, and the left-hand side arrow shows the new right atrial lead.

Under general anesthesia, three separate punctures from the left axillary vein were obtained. An attempt to introduce the right ventricular lead into the right ventricular apex in the anteroposterior (AP) view failed, but advancing the right ventricular lead (Medtronic CapSureFix^®^) into the right anterior oblique (RAO) 30^˚^ view proved easy. Pacing the lead confirmed a good ventricular capture (capturing threshold = 0.75 × 0.5 Volt msec). The implantation of the coronary sinus lead was done using a peel-away long sheath while injecting through mandarin in the left anterior oblique (LAO) 30˚ projection. A suitable posterolateral branch was visualized. A 4-French dual cathodic Medtronic lead (Attain Ability®) was employed in order to advance the lead as far as possible, minimize the chance of the phrenic nerve stimulation, and maximize interventricular resynchronization (capturing threshold = 1 × 0.5 Volt msec). The coronary sinus lead having been secured, the right atrial lead (Medtronic CapSureFix®, 52 cm) implantation was done in RAO and LAO projections during pacing to find the best atrial capturing threshold (capturing threshold = 1 × 0.5 Volt msec) (Figure 2). After the leads were secured to the underlying pectoral tissue, they were connected to the pacemaker generator. 

**Figure 3:**
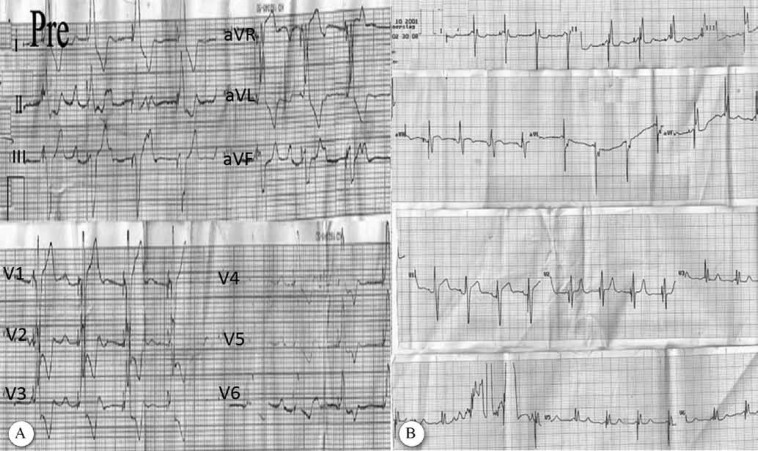
Electrocardiogram of the patient before (A) and after (B) cardiac resynchronization therapy device implantation with obvious QRS narrowing

Immediately after CRT device implantation, electrical resynchronization, manifested as a QRS duration of 80 msec, was achieved (Figure 3). One month later, exercise capacity increased and dyspnea on exertion decreased. Six months later, systemic ventricular ejection fraction rose from 35% to 40% with a decrease in end-systolic and diastolic dimensions.

## Discussion

Rodriguoz-Cruz et al.^4^ implanted a CRT device in a 22-year-old patient with CC-TGV, pulmonary atresia, and ventricular septal defect and reported improved systemic ventricular contractility and systolic blood pressure. Kakavand et al.^5^ (2006) implanted a CRT device for a 32-year-old patient with CC-TGV, ventricular septal defect, and acute heart failure and reported the immediate hemodynamic effects and improvement of symptoms. The largest retrospective study on CRT device implantation (in 11 CC-TGV patients among 60 patients with CHD) was published by Cechin et al.^6^ (2009), who reported a decrease in the median QRS width from 149 msec to 120 msec and an increase in the median ejection fraction from 36% to 42% (p value < 0.001). In that study, 8 out of 13 patients with a single-ventricle morphology had a “strong CRT response”, defined as either an improvement of symptoms and/or increased ventricular function by ≥ 10 ejection fraction units.

Regarding the coronary sinus anatomy in patients with CC-TGV, the most comprehensive study was conducted by Bottega et al.^7 ^(2009), who performed autopsy in 51 hearts with CC-TGV and found that 10 patients had an abnormal coronary sinus ostium. However, the most important distinction was that the ventricular veins were similar to the smaller epicardial veins that drain the right ventricle in patients without CHD.

Our patient had complete heart block and CC-TGV. As she grew up, her systemic right ventricle gradually failed against the aortic pressure and brought about heart failure symptoms. Despite the small size of her coronary sinus and its branches, the techniques mentioned above enabled us to implant a small coronary sinus lead.

We believe that appropriate patient selection, awareness of the technical challenges, and knowledge of the means and ways to overcome them are the three prerequisites for success in implanting CRT devices in CHD patients.

## Conclusion

Coronary computed tomographic (CT) angiography for the evaluation of the coronary sinus vein and its tributaries prior to CRT device implantation in CHD patients reduces the procedure time. What is more, CT angiography can diagnose abnormalities that may complicate the procedure (e.g. persistent left superior vena cava). Furthermore, different fluoroscopic projections other than the AP view (i.e. deep RAO or left lateral views) can help find the retrosternal chamber more easily (the morphological right ventricle in our patient). The use of contrast rather than deflectable electrophysiology catheters is recommended for the cannulation of the coronary sinus ostium. The availability of multiple coronary sinus leads (particularly those with small calibers) enables the operator to advance the lead as for as possible and, thus, maximize resynchronization and minimize the chance of the phrenic nerve stimulation.

## Notes:

***This paper should be cited as:*** Mollazadeh R, Eslami M. Cardiac Resynchronization Therapy Device Implantation in a Patient with Congenitally Corrected Transposition of Great Vessels. J Teh Univ Heart Ctr 2017;12(1):35-38.
